# AC-YOLO: A lightweight ship detection model for SAR images based on YOLO11

**DOI:** 10.1371/journal.pone.0327362

**Published:** 2025-07-30

**Authors:** Rui He, Dezhi Han, Xiang Shen, Bing Han, Zhongdai Wu, Xiaohu Huang

**Affiliations:** 1 College of Information Engineering, Shanghai Maritime University, Shanghai, China; 2 School of Computer Science, The University of Sydney, Sydney, New South Wales, Australia; 3 Shanghai Ship and Shipping Research Institute Co., Ltd., Shanghai, China; Wuhan University of Technology, CHINA

## Abstract

Synthetic Aperture Radar (SAR), renowned for its all-weather monitoring capability and high-resolution imaging characteristics, plays a pivotal role in ocean resource exploration, environmental surveillance, and maritime security. It has become a fundamental technological support in marine science research and maritime management. However, existing SAR ship detection algorithms encounter two major challenges: limited detection accuracy and high computational cost, primarily due to the wide range of target scales, indistinct contour features, and complex background interference. To address these challenges, this paper proposes AC-YOLO, a novel lightweight SAR ship detection model based on YOLO11. Specifically, we design a lightweight cross-scale feature fusion module that adaptively fuses multi-scale feature information, enhancing small target detection while reducing model complexity. Additionally, we construct a hybrid attention enhancement module, integrating convolutional operations with a self-attention mechanism to improve feature discrimination without compromising computational efficiency. Furthermore, we propose an optimized bounding box regression loss function, the Minimum Point Distance Intersection over the Union (MPDIoU), which establishes multi-dimensional geometric metrics to accurately characterize discrepancies in overlap area, center distance, and scale variation between predicted and ground truth boxes. Experimental results demonstrate that, compared with the baseline YOLO11 model, AC-YOLO reduces parameter count by 30.0% and computational load by 15.6% on the SSDD dataset, with an average precision (AP) improvement of 1.2%; on the HRSID dataset, the AP increases by 1.5%. This model effectively reconciles the trade-off between complexity and detection accuracy, providing a feasible solution for deployment on edge computing platforms. The source code for the AC-YOLO model is available at: https://github.com/He-ship-sar/ACYOLO.

## 1 Introduction

Synthetic Aperture Radar (SAR) operates by actively transmitting microwave signals and receiving backscatter echoes from surface targets, enabling the reconstruction of high-spatial-resolution two-dimensional images [1]. Due to the ability of microwaves to penetrate clouds and SAR’s all-weather imaging capability, it offers distinct advantages in dynamic ocean monitoring. Specifically, SAR quantifies differences in dielectric constants between targets and the sea surface, effectively capturing the scattering characteristics of metallic ship structures against ocean backgrounds. Furthermore, its multi-mode imaging mechanisms (e.g., spotlight, stripmap) accommodate diverse resolution requirements, providing multi-scale data support for ship detection [2]. With the rapid expansion of the global maritime economy, SAR has emerged as a critical infrastructure for real-time ship monitoring, illegal fishing detection, and maritime rescue operations. The accuracy and efficiency of SAR-based ship detection directly impact the timeliness and reliability of maritime security and law enforcement [3].

Despite SAR’s significant advantages in complex sea conditions, traditional SAR ship detection methods suffer from inherent limitations rooted in their physical and mathematical modeling frameworks. First, nonlinear scattering effects induced by dynamic sea surfaces—such as Bragg resonance and wind-wave modulation—result in non-stationary coupling between target echoes and sea clutter in the time-frequency domain. Consequently, statistical model-based Constant False Alarm Rate (CFAR) detectors are highly susceptible to threshold drift, leading to an exponential increase in false alarm rates when sea state levels exceed grade 3 [4]. Second, nearshore scenarios are complicated by strong scatterers like islands and drilling platforms, causing spatial aliasing effects. Additionally, SAR’s side-looking imaging geometry introduces layover and shadow distortions, significantly undermining the geometric separability of targets [5]. Experimental studies [6] indicate that when the radar cross-section (RCS) difference between ships and the background is less than 5 dB, traditional detection probabilities drop below 60%. A more pressing challenge arises from the coexistence of ship multi-scale characteristics and irregular motion patterns (e.g., variable speed, turning), necessitating detection algorithms with wide dynamic range adaptability, sub-pixel localization precision, and millisecond-level response—requirements that conventional physics-based frameworks struggle to meet due to their limited feature representation capacity and excessive computational redundancy [7].

The adaptive feature extraction capability of deep learning represents a paradigm shift in overcoming SAR ship detection bottlenecks. By constructing differentiable mappings from low-level scattering features to high-level semantics, recent studies have elevated detection accuracy to the object level [8]. Notably, the nonlinear fitting capability of deep neural networks to speckle noise compensates for the inadequacies of statistical models reliant on prior clutter distribution assumptions, driving SAR target detection toward an end-to-end intelligent framework. Based on network architecture, deep learning detection methods are broadly categorized into two-stage and one-stage frameworks:

Two-stage detectors generate candidate regions using a region proposal network (RPN), followed by classification and regression refinement. Ren *et al*. [9] proposed merging RPN with Fast R-CNN [10] to address efficiency issues inherent in stepwise training, achieving end-to-end optimization with enhanced detection speed. However, these methods still suffer from slow runtime and high computational complexity, hindering real-time applications. Building upon this, He *et al*. [11] augmented the Faster R-CNN [9] framework by incorporating a mask prediction branch, overcoming the limitations of separating detection and instance segmentation tasks, thereby enabling efficient multi-task optimization. While two-stage detectors exhibit high accuracy and robust semantic understanding, their high complexity limits their suitability for real-time scenarios. To mitigate efficiency bottlenecks, one-stage detectors reformulate detection as a classification and regression problem. Liu *et al*. [12] introduced the SSD framework, discretizing the bounding box output space into default boxes with varied scales and aspect ratios, achieving notable speed and accuracy gains. However, its reliance on predefined anchor boxes introduces parameter sensitivity and complex hyperparameter tuning. Addressing this, Tian *et al*. [13] proposed the FCOS detector, eliminating anchor boxes via per-pixel prediction, reducing computational complexity and dependency on hyperparameters, and achieving higher accuracy with a simpler structure. While early one-stage models slightly lagged behind two-stage counterparts in accuracy, continuous architectural innovations have narrowed this gap, particularly in SAR target detection.

Beyond traditional feature-based frameworks frameworks, researchers have explored speckle-resistant feature enhancement approaches to enhance SAR ship detection performance. Dellinger *et al*. [14] developed the SAR-SIFT algorithm, which improves gradient definition and feature matching to counteract performance degradation caused by speckle noise, thereby enhancing the robustness of multi-resolution image registration. However, SAR image ship detection still faces challenges such as imaging noise interference, complex backgrounds, and small target omission. To tackle these issues, Wang *et al*. [15] introduced the Non-Local Channel Attention Network (NLCANet), modeling pixel-region correlation and channel dependency to mitigate weakened target features in complex backgrounds. Hu *et al*. [16] proposed the Feature Interaction Network (FINet), integrating object-level and pixel-level information to improve localization precision under speckle noise and clutter interference, though its small target detection capability remains limited. Building on these advancements, Huang *et al*. [17] incorporated Efficient Channel Attention (ECA) and Swin Transformer modules into the backbone of the EST-YOLOv5s model, enhancing small target detection accuracy. Zhang *et al*. [18] introduced a Class Imbalance Loss (CI Loss) to alleviate performance bias caused by class distribution imbalance via gradient redistribution. Yet, multi-scale feature differences and sample representativeness in complex environments require further optimization. In response to this, Gong *et al*. [19] incorporated Context Attention Modules (CAM), Scale Enhancement Modules (SEM), and Scale Selection Modules (SSM), employing weighted negative sampling to enhance small target features and sample representativeness, thereby improving detection performance in complex maritime scenarios.

Despite the significant progress achieved by deep learning-based SAR ship detection models, several critical challenges persist. SAR images are highly susceptible to speckle noise and sea clutter interference, particularly affecting the detection of small targets such as fishing boats. Current models, constrained by limited receptive fields, struggle to capture global context information, leading to compromised detection stability and robustness. Furthermore, ships in SAR imagery exhibit substantial scale variations (e.g., cargo ships vs. small boats), and conventional Feature Pyramid Networks (FPN) may fail to effectively fuse multi-scale features, increasing the likelihood of small target omission. In addition, deep learning models generally demand high computational complexity and large parameter counts, posing challenges for deployment on resource-constrained edge platforms such as shipborne or satellite-based systems. Structural optimizations are necessary to balance accuracy and efficiency while maintaining real-time performance. Moreover, densely packed ships in SAR images often share similar aspect ratios but vary significantly in size. Conventional Intersection over Union(IoU) loss functions (e.g., GIoU, DIoU) rely primarily on center point distance and aspect ratio for regression optimization, leading to localization inaccuracies that hinder the precise detection of densely arranged targets. Addressing these challenges is crucial for the advancement of SAR ship detection methodologies and their practical deployment in maritime monitoring applications.

To address the challenges of SAR ship detection in complex maritime environments, we propose AC-YOLO, a lightweight and efficient detection model that integrates multiple novel designs to achieve high accuracy with low computational cost. The key innovations are as follows:

- We designed a hybrid attention mechanism named ACmix, which merges the advantages of convolution and self-attention. In this module, input features are projected into parallel paths via shared 1×1 convolutions, and learnable scalar parameters are introduced to dynamically balance the outputs of the convolutional and attention branches. This design effectively combines global context awareness with local detail preservation, thereby improving small object detection accuracy by 1.4% without increasing computational complexity.
- To enhance multi-scale feature fusion while maintaining model compactness, we developed the Cross-Channel Feature Mixing (CCFM) module. This module utilizes lightweight fusion blocks that perform element-wise addition between adjacent feature scales, significantly reducing redundant computation. Compared to conventional feature fusion strategies, CCFM achieves a 30.2% reduction in model parameters with negligible accuracy loss, which facilitates efficient deployment on edge devices.
- We proposed a novel loss function, Minimum Point Distance Intersection over the Union (MPDIoU), to improve bounding box regression in scenarios where predicted and ground truth boxes have similar aspect ratios but differ in scale. Unlike traditional IoU-based losses, MPDIoU minimizes the Euclidean distance between the top-left and bottom-right corners of the predicted and ground truth boxes, requiring only the computation of four coordinate points. This simplification enhances localization precision and computational efficiency.
- Extensive experiments conducted on two benchmark SAR ship datasets, SSDD [20] and HRSID [21], demonstrate that AC-YOLO outperforms 13 state-of-the-art detection models. Our model achieves superior accuracy while maintaining a lightweight architecture with fewer parameters and lower FLOPs, making it particularly suitable for real-time applications in resource-constrained environments such as edge computing.

This paper is organized into five sections. Sect 1 introduces the background, motivation, and challenges of research in SAR ship detection. Sect 2 provides a comprehensive review of related work, summarizing recent advancements and identifying existing limitations. Sect 3 presents a detailed description of the proposed AC-YOLO model, including its architectural design and key technical innovations. Sect 4 outlines the experimental setup, describes dataset selection, evaluation metrics, and implementation details, followed by an in-depth performance analysis. Sect 5 summarizes the key findings, highlights the contributions of this study, and discusses potential directions for future research.

## 2 Related work

### 2.1 Traditional ship detection algorithms

Traditional SAR ship detection methods have predominantly relied on CFAR algorithms due to their capability for dynamic threshold adjustment. However, their performance is limited by the non-uniformity of sea clutter distributions and the diversity of target scales. Researchers have enhanced detection accuracy and robustness by integrating physical models with adaptive strategies. Zhu *et al*. [22] proposed a novel hierarchical Ship Detection from Spaceborne Optical Imagery (SDSOI) method based on shape and texture features, addressing the limitations of existing SAR-based methods in terms of limited sensor availability, long revisit cycles, and low resolution, which result in poor real-time performance and high false alarm rates. Nevertheless, SDSOI’s ability to resolve multiple targets in complex sea states remains inadequate. To mitigate this limitation, Tello *et al*. [23] introduced a wavelet-transform-based ship detection method for SAR images, leveraging statistical behavioral differences between ships and surrounding seas through wavelet coefficient interpretation, offering more reliable detection. However, its computational robustness under complex clutter interference still requires improvement. Addressing this, Gao *et al*. [24] proposed an adaptive and fast CFAR algorithm based on automatic censoring (AC), G0-distributed clutter modeling, and clustering methods, enhancing robust target detection and real-time performance in high-resolution SAR images under challenging clutter conditions (e.g., non-uniform regions, multi-target interference, and clutter edges).

While traditional ship detection approaches initially dominated due to their theoretical transparency and low resource consumption, their rigidity and computational inefficiency have become significant drawbacks in high-resolution and complex scene applications. These limitations have accelerated the adoption of deep learning techniques, which now dominate SAR ship detection methodologies [25].

### 2.2 Deep learning-based SAR ship detection algorithms

In recent years, deep learning advancements have driven significant breakthroughs in SAR ship detection [26], forming two mainstream technical routes centered around two-stage and one-stage detection frameworks. Two-stage methods, based on region proposal networks, achieve precise localization through cascaded feature processing, whereas one-stage methods leverage end-to-end architectures to excel in detection efficiency. Together, they propel SAR target detection toward higher accuracy and faster response times.

#### 2.2.1 Two-stage deep learning-based SAR ship detection algorithms

Compared to traditional methods, two-stage deep learning-based SAR ship detection algorithms offer superior detection accuracy and interference resilience. Their advantages in distributed optimization, multi-scale adaptability, and semi-supervised compatibility have established them as mainstream solutions in SAR ship detection. Zhang *et al*. [27] proposed the Center-Head Point Detector (CHPDet), leveraging rotational Gaussian heatmaps and orientation-invariant feature learning to address the angular prediction bias, computational redundancy, and prior size utilization limitations in existing rotated anchor-based methods. However, CHPDet still exhibits limitations in multi-target detection under complex sea conditions, particularly in high-density target regions. Building on this, Gao *et al*. [28] introduced the Attention-free Global Multi-Scale Fusion Network (AGMF-Net), employing a spatial bias module to construct global perception, multi-task feature enhancement, and context-decoupled detection mechanisms, effectively handling background interference and small target detection coupling in remote sensing images. Nonetheless, AGMF-Net still requires improvements in handling extreme scale variations and real-time constraints. Furthermore, Li *et al*. [29] proposed the Multi-dimensional Calibration and Suppression Network (MCSNet), which innovatively integrates SAR-RFI joint modeling, multi-dimensional feature calibration, and residual detail recovery techniques. This approach effectively preserves scene scattering characteristics and enhances SAR image quality.

Although two-stage methods exhibit strong feature learning and high precision, their high computational complexity restricts real-time applicability. Consequently, selecting appropriate detection algorithms depends critically on specific operational requirements.

#### 2.2.2 One-stage deep learning-based SAR ship detection algorithms.

One-stage ship detection algorithms primarily include the You Only Look Once (YOLO) series [30], SSD [12], and RetinaNet [31]. Among them, the YOLO family adopts a grid-based prediction mechanism, unifying localization and classification within a single network, significantly enhancing detection speed, and making it well-suited for real-time SAR ship detection scenarios.

Since its inception, the YOLO series has demonstrated unique advantages in SAR ship detection, particularly in multi-scale feature fusion, rotation-invariant target detection, and noise robustness. For instance, YOLOv4 [32] introduces a bidirectional feature pyramid network to strengthen small-scale target detection by aggregating features across layers, effectively addressing target scale variations in SAR images. However, it struggles with accurate angle prediction when dealing with arbitrary-oriented ship targets. To tackle this, Han *et al*. [33] incorporated an angular parameter into the detection head and adopted circular smooth labels to resolve periodic ambiguities, enhancing rotational target detection accuracy. Nonetheless, inherent speckle noise in SAR images still degrades detection performance. Addressing this, Amrani *et al*. [34] proposed an improved YOLO model that integrates attention mechanisms to suppress noise-induced feature responses, enhancing model robustness.

Recently, researchers have introduced various innovative modifications tailored to the complexity of SAR ship detection. In feature fusion, Zhang *et al*. [35] combined multi-modal features from polarimetric SAR data with conventional RGB features, employing cross-modal interaction to improve detection robustness in challenging sea conditions. In terms of lightweight design, Jiang *et al*. [36] utilized structural re-parameterization and modular design to reduce model parameters while maintaining detection accuracy, and facilitating deployment on edge devices. Additionally, Tranheden *et al*. [37] introduced a cross-domain transfer learning framework using pseudo-sample generation, effectively alleviating model degradation under low-sample conditions.

Despite these improvements, one-stage detectors often face challenges in balancing lightweight design with effective multi-scale feature fusion. Compressing network depth or feature layers to reduce computational load can impair small target detection, especially under complex sea clutter backgrounds, leading to feature loss and localization deviations. To address this, we propose a novel SAR ship detection model, AC-YOLO, based on the YOLO11 framework, specifically optimized for SAR imagery characteristics to achieve superior accuracy-efficiency trade-offs.

## 3 Methodology

### 3.1 Baseline model: YOLO11

**Fig 1:**
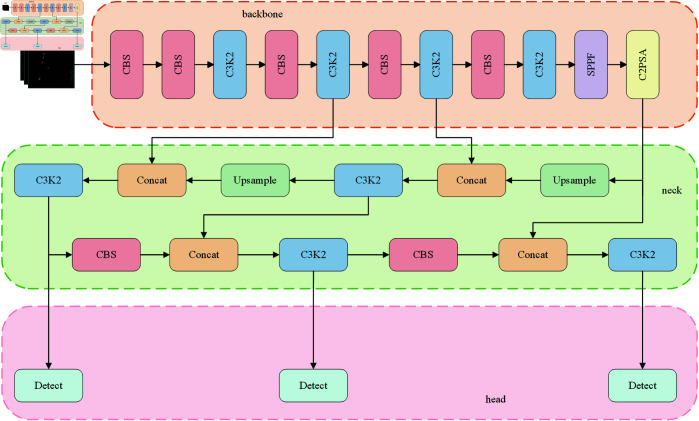
YOLO11 network architecture.

YOLO11 represents the latest advancement in the YOLO series, achieving significant improvements in backbone, neck, and detection head components compared to its predecessor YOLOv8 [38]. It has demonstrated outstanding performance in computer vision object detection tasks and is currently considered one of the state-of-the-art (SOTA) models. Given its remarkable achievements, YOLO11 is selected as the baseline model in this study. The overall architecture of YOLO11 comprises three core components: the backbone network, which serves as the fundamental feature extractor; the neck network, responsible for enhancing the extracted features; and the detection head, tasked with final object detection prediction. The structure of YOLO11 is illustrated in Fig 1.

**Fig 2:**
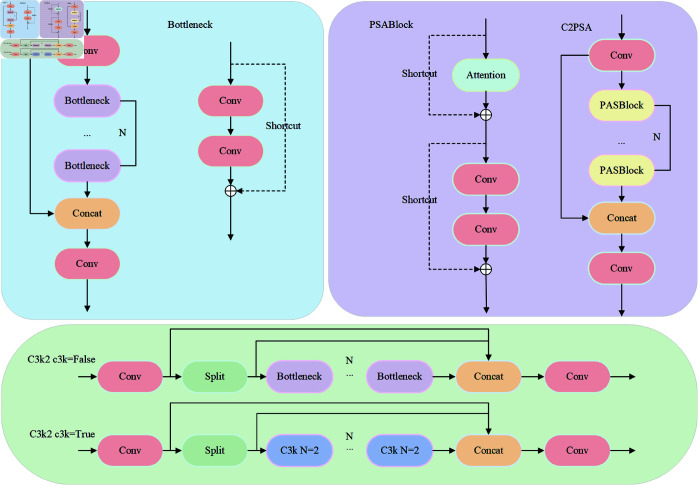
Structures of C2PSA and C3K2 modules.

In the backbone, YOLO11 introduces the C3K2 and C2PSA modules. The C3K2 module, parameterized as c3k, is configured with c3k set to False in the shallow layers, resembling the C2f module in YOLOv8. It optimizes information flow by splitting feature maps and applying a series of smaller 3×3 convolution kernels. The C2PSA module embeds a multi-head attention mechanism within the C2 block, refining the network’s ability to selectively focus on regions of interest via spatial attention applied to extracted features. The detailed structure of C2PSA and C3K2 is shown in Fig 2.

For feature enhancement, YOLO11 retains the Spatial Pyramid Pooling - Fast (SPPF) module from YOLOv8. This module aggregates features at multiple scales, enhancing the network’s capability to detect objects of varying sizes.

Regarding the detection head, YOLO11 incorporates two Depthwise Separable Convolutions (DWConv) in the classification and detection branches to reduce parameter count and computational cost. DWConv is an efficient convolutional operation designed to minimize complexity and resource usage.

While YOLO11 surpasses prior models in both accuracy and parameter efficiency, SAR ship detection tasks still face challenges such as missed detections, false alarms, and lightweight deployment constraints. To this end, we propose an enhanced SAR ship detection model, AC-YOLO, based on YOLO11, specifically optimized for the unique characteristics of SAR imagery. AC-YOLO aims to reduce model parameters and detection errors while comprehensively improving detection reliability and efficiency.

### 3.2 Proposed model: AC-YOLO

**Fig 3:**
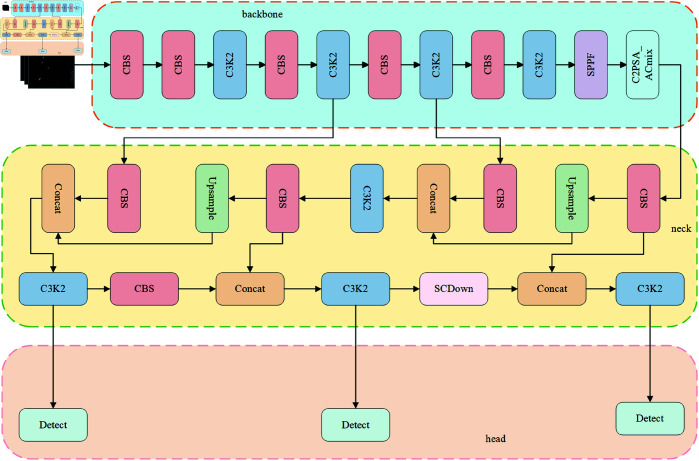
AC-YOLO model architecture.

The architecture of the proposed AC-YOLO model is illustrated in Fig 3. It consists of three key components: an improved backbone network, a cross-scale feature fusion neck, and an optimized detection head.

In the backbone, we integrate the ACmix module to replace the original C2PSA structure in YOLO11. This module fuses self-attention and convolutional operations, effectively combining global context awareness with local detail preservation.

In the neck, we design a lightweight CCFM module to integrate multi-scale features via fusion blocks, reducing computational redundancy and improving feature extraction efficiency. This design achieves a 30.2% reduction in parameter count while ensuring efficient multi-scale feature interaction.

Considering the densely arranged nature of ships in SAR images, we introduce the MPDIoU loss function. By minimizing the distance between the top-left and bottom-right corners of predicted and ground truth boxes, MPDIoU mitigates the localization errors accumulated by the aspect ratio coupling in conventional IoU-based loss functions, thus enhancing detection accuracy in dense target scenarios.

### 3.3 Self-attention and convolution fusion module

We introduce the ACmix [39] module and embed it into the C2PSA mechanism of YOLO11. This module leverages a unified computation framework to seamlessly integrate the advantages of self-attention and convolution, enabling efficient modeling of both global and local features. The core design of ACmix comprises three key components: a shared projection layer, dual-path parallel computation, and an adaptive fusion gate.

The shared projection layer employs linear transformations to map input features into query, key, and value (*Q*,*K*,*V*) components for the self-attention path, as well as dynamic kernel parameters for the convolution path. This design allows parameter sharing, reducing projection computation by 30%. In dual-path computation, the self-attention path captures long-range spatial dependencies via a multi-head mechanism, addressing the limited receptive field problem of convolution. Simultaneously, the convolution path utilizes differentiable spatial shift operations to extract local structural features, preserving fine-grained details. The adaptive fusion gate uses a sigmoid function to generate a spatially-aware weighting map (α∈[0,1]), dynamically balancing the outputs of both paths—enhancing global modeling in texture-rich regions while reinforcing local feature extraction in smooth regions.

**Fig 4:**
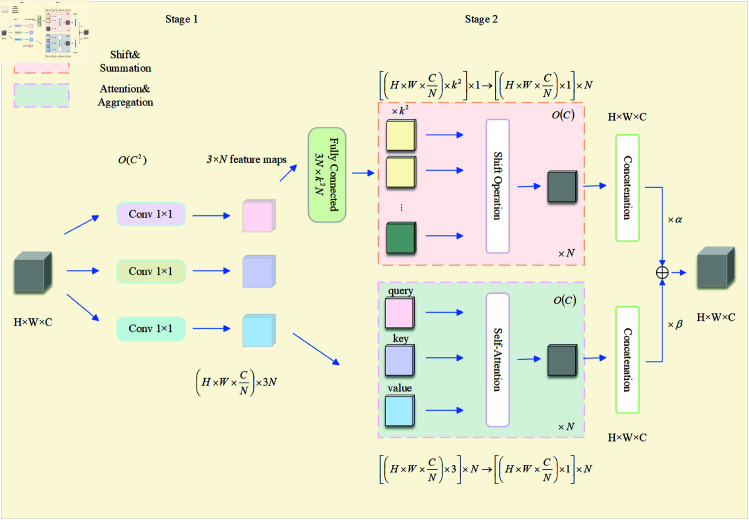
ACmix module architecture.

The ACmix module not only maintains lightweight characteristics but also ensures efficient fusion of global-local features through mathematically equivalent shared projections and differentiable shift operations. This makes it particularly suitable for high-precision tasks such as small object detection in remote sensing imagery and medical image segmentation. The structure of the ACmix module is depicted in Fig 4 and its computational details are provided in Table 1.

**Table 1:** The computational flow of the C2PSA_ACmix fusion module is defined as follows.

Module C2PSA_ACmix
*Input*: (*B*,*C*1,*H*,*W*)
*Conv1* (1×1):(B,2*C,H,W)
*Split into a*(*B*,*C*,*H*,*W*) *and b*(*B*,*C*,*H*,*W*)
*Process b through n PSABlocks*: (B,C,H,W)→…→(B,C,H,W)
*Concatenate a and processed b*: (*B*,2*C,*H*,*W*)
*Conv2* (1×1):(B,C1,H,W)
*Output*: (*B*,*C*1,*H*,*W*)

### 3.4 Cross-channel feature mixing module

In real-time object detection tasks, the YOLO series typically relies on stacked convolutional layers and strode downsampling to construct multi-scale feature pyramids. However, such designs inherently trade-off between speed and accuracy: frequent striding leads to loss of fine-grained features, while insufficient receptive field expansion in deeper layers limits the network’s ability to capture long-range context relationships. This issue becomes particularly severe in scenarios involving densely packed small targets, where fixed-scale convolution kernels fail to dynamically adapt to varying target distributions, resulting in increased missed detections.

Inspired by DETR series models, we propose the CCFM [40] to address these challenges within a real-time detection framework. This module employs a lightweight channel interaction mechanism and a dynamic convolution kernel generation strategy to enhance multi-scale feature fusion efficiency without introducing additional computational overhead. The CCFM module consists of two critical components: a multi-scale feature alignment layer and channel-aware dynamic convolution.

**Fig 5:**
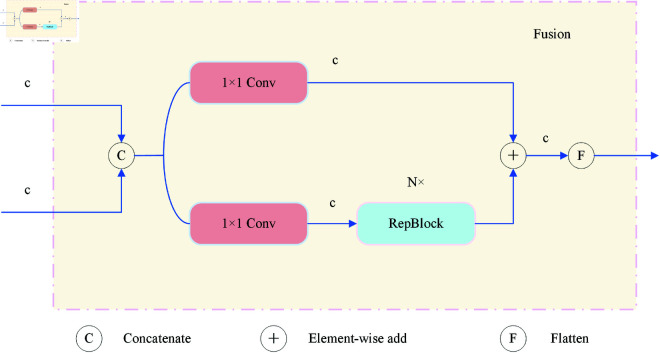
CCFM module architecture.

The multi-scale feature alignment layer adopts a spatial pyramid pooling structure, decomposing input feature maps into sub-regions of varying granularity (e.g., 1×1, 3×3, 5×5 grids). It extracts both local and global statistics through parallel max-pooling and average-pooling operations. The resulting feature descriptors undergo channel compression via point-wise convolutions, forming compact context priors. The channel-aware dynamic convolution module utilizes the prior information to generate weight matrices that dynamically adjust convolution kernel parameters. Specifically, fully connected layers predict attention coefficients for each channel, which are then used to perform weighted fusion along the channel dimension. This mechanism allows the convolution operation to adapt its receptive field based on semantic characteristics, enhancing sensitivity to long-tail targets while preserving high-frequency details. The overall structure of the CCFM module is illustrated in Fig 5.

### 3.5 MPDIoU loss function

In YOLO11, the detection task loss function comprises three parts: object confidence loss, classification loss, and bounding box regression loss. The overall loss function is expressed as Eq (1):

$$Loss=Loss_{obj}+Loss_{cls}+Loss_{box}$$ (1)

Bounding box regression loss (*Loss*_*box*_) is a key factor influencing detector performance. Conventional IoU-based regression losses, including IoU, GIoU, DIoU, and CIoU, incorporate geometric factors such as overlap area, center point distance, and aspect ratio to calculate the loss.

The traditional Intersection over Union (IoU) metric is defined as the ratio of the intersection area to the union area between the predicted bounding box and the ground truth bounding box. The formula is given by Eq (2):

$$IoU=\frac{{ℬ}_{gt}\cap {ℬ}_{prd}}{{ℬ}_{gt}\cup {ℬ}_{prd}},$$ (2)

where *B*_*gt*_ and *B*_*prd*_ represent the ground truth and predicted bounding boxes, respectively.

Complete IoU (CIoU) extends the traditional IoU by incorporating the center point distance and aspect ratio consistency. The CIoU formula is expressed as Eq (3):

$$CIoU=IoU-\frac{\rho^{2}\left(B_{gt},B_{prd}\right)}{C^{2}}-\alpha V$$ (3)

where $\rho^{2}$ denotes the squared Euclidean distance between the center points of *B*_*gt*_ and *B*_*prd*_, and C represents the diagonal length of the smallest enclosing convex hull that contains both the ground truth and predicted bounding boxes. The variable V quantifies the consistency of the aspect ratio between the predicted and ground truth boxes and is defined as Eq (4):

$$V=\frac{4}{\pi^{2}}{\left(\arctan\frac{w^{gt}}{h^{gt}}-\arctan\frac{w^{prd}}{h^{prd}}\right)}^{2}$$ (4)

where ${\text{w}}^{\text{gt}}$, ${\text{h}}^{\text{gt}}$, ${\text{w}}^{\text{prd}}$ and ${\text{h}}^{\text{prd}}$ represent the width and height of the ground truth and predicted bounding boxes, respectively. The weighting parameter α is computed as Eq (5):

$$\alpha =\frac{V}{1-IoU+V}$$ (5)

However, these methods exhibit inherent limitations. Specifically, when the predicted and ground truth boxes do not overlap, IoU-based losses fail to provide valid gradient information, leading to optimization difficulties. Additionally, fixed penalty terms for aspect ratios are inadequate for handling extreme-scale targets, such as elongated or flattened objects. Moreover, simultaneous consideration of multiple geometric factors may introduce conflicting optimization directions, adversely affecting model convergence—particularly problematic for small objects or densely packed scenes, where localization precision is crucial.

To overcome these challenges, we propose the MPDIoU [41], a novel bounding box similarity metric, and define a corresponding regression loss function. MPDIoU considers overlapping and non-overlapping regions, center point distance, and width-height deviations while simplifying the computational process. The MPDIoU formulation is as follows Eq (6):

$$MPDIoU=\frac{A\cap B}{A\cup B}-\frac{d_{1}^{2}}{w^{2}+h^{2}}-\frac{d_{2}^{2}}{w^{2}+h^{2}}$$ (6)

In the given formulas, A represents the area of the predicted bounding box, while B denotes the area of the ground truth bounding box. The terms $d_{1}^{2}$ and $d_{2}^{2}$ correspond to the squared Euclidean distances between the top-left and bottom-right corners of the predicted and ground truth bounding boxes, respectively. Additionally, *w* and *h* represent the width and height of the input image.The intersection and union areas between the predicted and ground truth bounding boxes are computed as Eq (7):

$$A\cap B=max(0,\min(x_{2A},x_{2B})-\max(x_{1A},x_{1B}))\times \max(0,\min(y_{2A},y_{2B})-\max(y_{1A},y_{1B}))$$ (7)

A∪B=A+B−A∩B (8)

The squared distances between the top-left and bottom-right corner points are given by:

$$d_{1}^{2}=(x_{1}^{B}-x_{1}^{A}{)}^{2}+(y_{1}^{B}-y_{1}^{A}{)}^{2}$$ (9)

$$d_{2}^{2}=(x_{2}^{B}-x_{2}^{A}{)}^{2}+(y_{2}^{B}-y_{2}^{A}{)}^{2}$$ (10)

Based on the MPDIoU metric, we define a novel bounding box regression loss function as follows:

$${ℒ}_{MPDIoU}=1-MPDIoU$$ (11)

**Fig 6:**
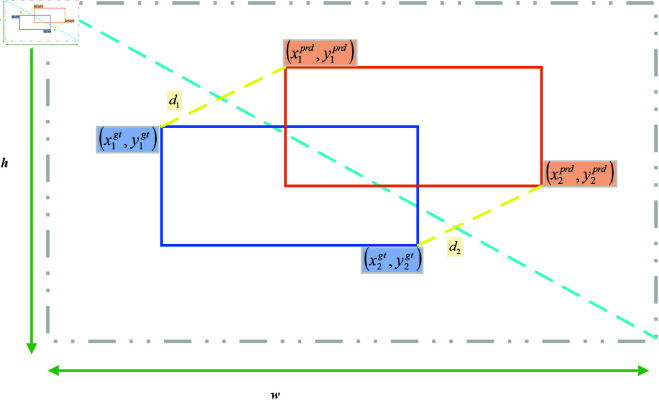
Overview diagram of MPDIoU.

This loss function optimizes bounding box regression by minimizing MPDIoU, ensuring effective differentiation between predicted and ground truth boxes in all cases. The schematic diagram of MPDIoU is shown in Fig 6.

The algorithmic flow of MPDIoU is outlined in Tables 2 and 3.

**Table 2:** Intersection over union with minimum points distance.

Input Two arbitrary convex shapes: $A,B\subseteq S\in {\mathbb{R}}^{n}$, width and height of input image: *w*,*h*.
Output MPDIoU
1. For A and B, $\left(x_{1}^{A},y_{1}^{A}),(x_{2}^{A},y_{2}^{A}\right)$ denote the top-left and bottom-right point coordinates of A,
$\left(x_{1}^{B},y_{1}^{B}),(x_{2}^{B},y_{2}^{B}\right)$ denote the top-left and bottom-right point coordinates of B.
2. $d_{1}^{2}=(x_{1}^{B}-x_{1}^{A}{)}^{2}+(y_{1}^{B}-y_{1}^{A}{)}^{2},\;d_{2}^{2}=(x_{2}^{B}-x_{2}^{A}{)}^{2}+(y_{2}^{B}-y_{2}^{A}{)}^{2}$
3. $MPDIoU=\frac{A\cap B}{A\cup B}-\frac{d_{1}^{2}}{w^{2}+h^{2}}-\frac{d_{2}^{2}}{w^{2}+h^{2}}$

**Table 3:** IoU and MPDIoU as bounding box losses.

Input Predicted *B*_*prd*_ and ground truth *B*_*gt*_ bounding box coordinates.
$B_{prd}=(x_{1}^{prd},y_{1}^{prd},x_{2}^{prd},y_{2}^{prd})$, $B_{gt}=(x_{1}^{gt},y_{1}^{gt},x_{2}^{gt},y_{2}^{gt})$, width and height of input image: *w*,*h*.
Output ${ℒ}_{IoU},{ℒ}_{MPDIoU}$
1. For the predicted box *B*_*prd*_, ensuring $x_{2}^{prd}>x_{1}^{prd}$ and $y_{2}^{prd}>y_{1}^{prd}$.
2. $d_{1}^{2}=(x_{1}^{prd}-x_{1}^{gt}{)}^{2}+(y_{1}^{prd}-y_{1}^{gt}{)}^{2},\;d_{2}^{2}=(x_{2}^{prd}-x_{2}^{gt}{)}^{2}+(y_{2}^{prd}-y_{2}^{gt}{)}^{2}$
3. Calculating area of *B*_*gt*_ and *B*_*prd*_: $A^{gt}=(x_{2}^{gt}-x_{1}^{gt})\times (y_{2}^{gt}-y_{1}^{gt}),\;A^{prd}=(x_{2}^{prd}-x_{1}^{prd})\times (y_{2}^{prd}-y_{1}^{prd})$
4. Calculating intersection *I* between *B*_*prd*_ and *B*_*gt*_: $x_{1}^{I}=\max(x_{1}^{prd},x_{1}^{gt}),\;x_{2}^{I}=\min(x_{2}^{prd},x_{2}^{gt})$
$y_{1}^{I}=\max(y_{1}^{prd},y_{1}^{gt}),\;y_{2}^{I}=\min(y_{2}^{prd},y_{2}^{gt})\;I=\left\{ \begin{array}{cc} (x_{2}^{I}-x_{1}^{I})\times (y_{2}^{I}-y_{1}^{I}), & \text{if }x_{2}^{I}>x_{1}^{I},y_{2}^{I}>y_{1}^{I}\\ 0, & \text{otherwise.} \end{array}\right.$
5. $IoU=\frac{I}{u},where𝒰=A^{gt}+A^{prd}-I$.
6. $MPDIoU=IoU-\frac{d_{1}^{2}}{h^{2}+w^{2}}-\frac{d_{2}^{2}}{h^{2}+w^{2}}$.
7. ${ℒ}_{IoU}=1-IoU,\;{ℒ}_{MPDIoU}=1-MPDIoU$.

## 4 Experiments

### 4.1 Experimental environment and parameters

All experiments were conducted on a high-performance computing platform equipped with a 16 vCPU Intel(R) Xeon(R) Platinum 8352V processor with a base clock speed of 2.10 GHz, alongside an RTX 4090 GPU. The operating system is Ubuntu 22.04. The implementation utilizes PyTorch 2.5.1 built on Python 3.12, with CUDA 12.4 for GPU acceleration. The detailed training parameters are summarized in Table 4.

**Table 4:** Experimental training parameters.

Component	Configuration
Epochs	150
Image size	640×640
Batch size	16
Initial learning rate	0.007
Momentum	0.937
Workers	8

### 4.2 Datasets

To verify the reliability and robustness of the proposed model, experiments were conducted on two publicly available datasets with different spatial resolutions: SSDD [20] and HRSID [21]. Detailed dataset parameters are listed in Table 5.

**Table 5:** Dataset specifications.

Parameters	HRSID	SSDD
Satellite	Sentinel-1B, TerraSAR-X, TanDEM	RadarSat-2, TerraSAR-X, Sentinel-1
Polarization method	HH, VV, HV	HH, VV, VH, HV
Resolution (m)	0.5~3	1~15
Image size (pixel^2^)	800×800	217×214-526×646
Number of images	5604	1160
Total number of ship target	16951	2456

For consistency with the YOLO11 framework, label files from the original VOC-formatted datasets were converted to the required TXT format. Following related research [42], the SSDD dataset was randomly split into training and testing sets at an 8:2 ratio, while the HRSID dataset was divided into training, testing, and validation sets at a 7:2:1 ratio.

#### 4.2.1 SSDD dataset.

The SAR Ship Detection Dataset (SSDD) [20] is widely used for evaluating detection models. It comprises 1,160 SAR images provided by Sentinel-1, TerraSAR-X, and RadarSat-2 satellites, containing a total of 2,456 ship targets. The image resolution ranges from 1 to 15 meters, with varied polarization modes and complex maritime scenarios.

#### 4.2.2 HRSID dataset.

The High-Resolution SAR Image Dataset (HRSID) [21], released publicly by Su Hao from the University of Electronic Science and Technology of China in January 2020, is designed for ship detection, semantic segmentation, and instance segmentation in high-resolution SAR images. The dataset consists of 5,604 images collected from Sentinel-1 and TerraSAR-X satellites, containing 16,951 annotated ship targets, each image with a resolution of 800 × 800 pixels.

### 4.3 Evaluation metrics

The performance of the proposed model is evaluated using precision (P), recall (R), and average precision (AP). Precision refers to the percentage of correctly detected ships among all detections, while recall denotes the percentage of correctly detected ships among all ground truth targets. The definitions are as follows:

$$P=\frac{N_{TP}}{N_{TP}+N_{FP}}$$ (12)

$$R=\frac{N_{TP}}{N_{TP}+N_{FN}}$$ (13)

True Positives *N*_*TP*_ refer to the number of correctly detected ship targets, while False Positives *N*_*FP*_ represent erroneously detected ship targets, and False Negatives *N*_*FN*_ denote missed ship targets. The AP quantifies the detection accuracy within a single category and is computed as follows:

$$mAP=AP=\int_{0}^{1}P(R)dR$$ (14)

where P denotes precision and R represents recall.

Additionally, we calculate AP metrics at different IoU thresholds (${AP}_{50}$, ${AP}_{75}$) and for different object scales (${AP}_{S}$ for small, ${AP}_{M}$ for medium, and ${AP}_{L}$ for large targets), as summarized in Table 6. To further assess the model’s efficiency, we report the number of parameters, floating point operations per second (FLOPs) and model size.

**Table 6:** Evaluation metrics.

Metric	Meaning
P	Precision
R	Recall
AP	Average Precision
${AP}_{50}$	AP at IoU threshold 0.5
${AP}_{75}$	AP at IoU threshold 0.75
${AP}_{S}$	AP for small objects (area < 32^2^)
${AP}_{M}$	AP for medium objects (area $32^{2}\sim 96^{2}$)
${AP}_{L}$	AP for large objects (area > 96^2^)
FLOPs	Floating Point Operations
Params	Number of model parameters
Model size	Model storage size

### 4.4 Comparative methods

To validate the effectiveness of the proposed approach, we compare AC-YOLO with 13 popular SAR ship detection algorithms, including: Faster R-CNN [9], Cascade R-CNN [43], SSD [12], ATSS [44], YOLOX-tiny [45], RetinaNet [31], RTMDet [46], GFL [47], YOLO11n, TOOD [48], Mask R-CNN [11], YOLOv5, YOLOv10n [49].

**Fig 7:**
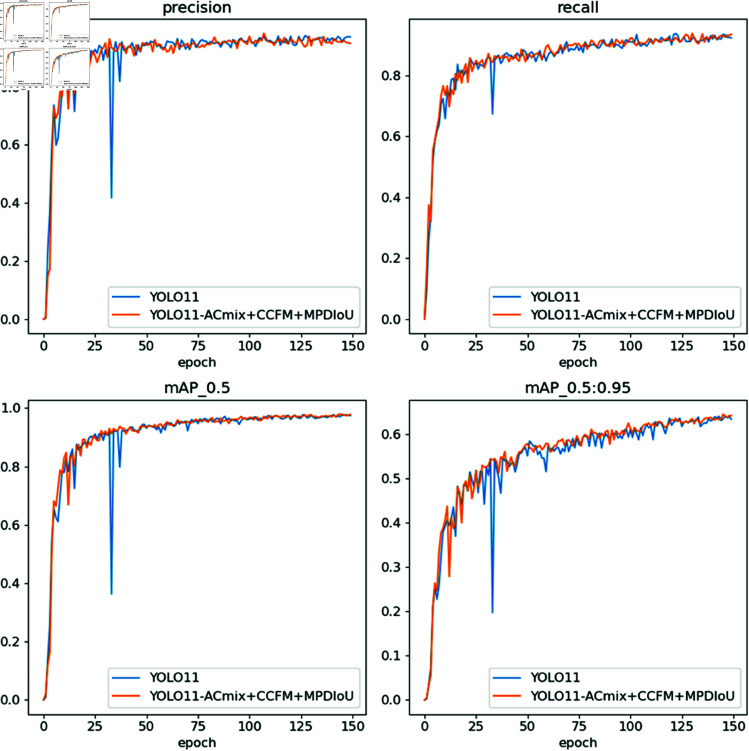
Precision, recall, and F1 score curves.

The four subplots respectively illustrate the variation trends of Precision, Recall, mAP at IoU=0.5, and mAP at IoU range 0.5–0.95 over 150 training epochs. The improved model demonstrates enhanced convergence speed, higher detection accuracy, and greater training stability compared to the baseline in Fig 7.

### 4.5 Comparative experiments

Table 7 details the experimental results of AC-YOLO compared to YOLO11 and other advanced models on SSDD and HRSID datasets.

**Table 7:** Comparative results on SSDD and HRSID datasets.

Methods	AP_50_(%)		FLOPs(G)	Params(M)	Model size(MB)
	SSDD	HRSID			
Faster R-CNN [9]	95.2	80.9	134	41.3	317.886
Cascade R-CNN [43]	93.8	84.3	209	69.2	531.664
SSD [12]	92.8	58.5	214	23.7	183.24
ATSS [44]	95.6	87.6	126	32.1	246.71
YOLOX-tiny [45]	95.6	88.6	7.571	5.0	68.65
RetinaNet [31]	79.3	79.0	177	36.3	278.586
RTMDet [46]	96.0	85.7	8.025	4.9	59.992
GFL [47]	93.2	87.9	177	32.3	245.823
YOLO11n	95.6	89.0	6.4	2.6	5.225
TOOD [48]	95.8	88.6	171	32.0	245.667
Mask R-CNN [11]	92.2	81.5	181	41.3	317.87
YOLOv5	96.5	86.8	7.2	2.5	5.032
YOLOv10n [49]	95.0	84.3	8.4	2.7	5.49
AC-YOLO	96.7	89.3	5.4	1.8	3.752

The results indicate that AC-YOLO achieves superior AP_50_ values of 96.7% and 89.3% on SSDD and HRSID datasets, respectively, outperforming the baseline YOLO11n and other models. Notably, AC-YOLO exhibits remarkable efficiency, with FLOPs reduced to 5.4G and parameter count compressed to 1.8M, significantly lower than most competitors, including lightweight models like YOLOv5. The model size is compressed to 3.752 MB, making it highly suitable for deployment on edge platforms and real-time applications.

**Fig 8:**
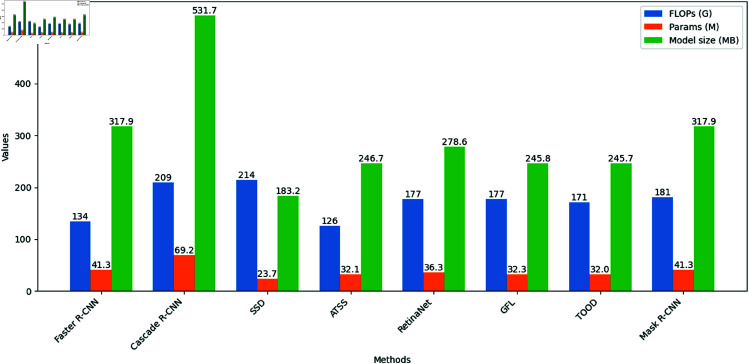
Comparison of different methods.

**Fig 9:**
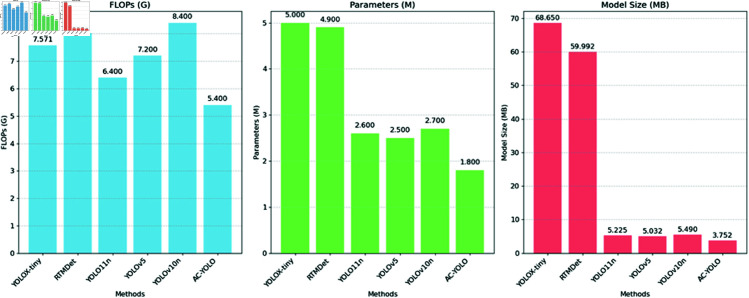
Comparison of different methods.

For a more intuitive comparison, we present bar charts comparing different models’ FLOPs, parameter counts, and model sizes in Figs 8 and 9.

We grouped the methods with comparable metric magnitudes into the same table for a more intuitive comparison. As shown in the table, the proposed AC-YOLO model outperforms the other models in terms of FLOPs, number of parameters, and model size.

### 4.6 Ablation study

To evaluate the individual contributions of each proposed module, we conducted ablation studies on both SSDD and HRSID datasets. The effectiveness of each component is validated by incrementally adding them to the baseline model and comparing performance metrics and model complexity.

In Table 8, the results demonstrate that the CCFM module plays a crucial role in reducing model parameters, lowering FLOPs, and minimizing model size. Specifically, compared to the baseline, the model parameter count decreased from 2.59 M to 1.815 M, FLOPs dropped from 6.4 G to 5.4 G, and the model size reduced from 5.225 MB to 3.752 MB. This confirms the effectiveness of the overall network design.

**Table 8:** Model complexity comparison in ablation study.

Baseline	ACmix	CCFM	MPDIoU	Params	FLOPs(G)	Model size (MB)
✓				2,590,035	6.4	5.225
✓	✓			2,598,859	6.4	5.239
✓		✓		1,806,803	5.4	3.738
✓	✓	✓		1,815,627	5.4	3.752
✓	✓	✓	✓	1,815,627	5.4	3.752

The results in Table 9 reveal that integrating ACmix, CCFM, and MPDIoU modules leads to optimal performance on the SSDD dataset. The AP improves from 61.7% to 62.9%, with AP_50_ reaching 96.7% and AP_75_ improving to 73.8%. In particular, the proposed model shows superior capability in detecting medium and large-scale targets, with AP_*M*_ and AP_*L*_ values achieving 71.9% and 73.1%, respectively. For small targets, the model maintains strong performance at 57.8%. Although not the highest, the performance remains at a relatively high level. The model achieves an accuracy of 71.9% for medium-sized objects AP_*M*_ and 73.1% for large-sized objects AP_*L*_, demonstrating a significant advantage in detecting medium and large objects.In summary, the integration of the ACmix, CCFM, and MPDIoU modules not only enhances the overall performance of the model but also exhibits superior performance across different IoU thresholds and object sizes. This fusion strategy effectively improves detection accuracy and robustness, enabling the model to maintain high performance across various detection tasks. Among all tested configurations, this combination yields the best results.

**Table 9:** Ablation results on SSDD dataset.

Baseline	ACmix	CCFM	MPDIoU	AP(%)	AP_50_(%)	AP_75_(%)	AP_*S*_(%)	AP_*M*_(%)	AP_*L*_(%)
✓				61.7	95.6	72.0	56.0	72.2	64.0
✓			✓	63.2	96.7	76.1	57.7	73.0	60.7
✓		✓		61.7	96.5	70.8	55.7	72.4	64.1
✓	✓			63.4	97.1	74.9	57.4	73.9	67.9
✓	✓	✓		62.2	97.1	71.5	56.2	72.9	64.7
✓	✓	✓	✓	62.9	96.7	73.8	57.8	71.9	73.1

The ablation experiments on the HRSID dataset further confirm the effectiveness of the proposed modules. The model’s AP increases from 65.9% to 67.4%, while AP_50_ improves from 89.0% to 89.3%, as shown in Table 10. These results highlight the robustness and stability of the proposed architecture across different datasets.

**Table 10:** Ablation results on HRSID dataset.

Baseline	ACmix	CCFM	MPDIoU	AP(%)	AP_50_(%)
✓				65.9	89.0
✓			✓	65.8	89.2
✓		✓		64.7	88.4
✓	✓			65.6	89.5
✓	✓	✓		65.0	88.8
✓	✓	✓	✓	67.4	89.3

### 4.7 Visualization analysis

**Fig 10:**
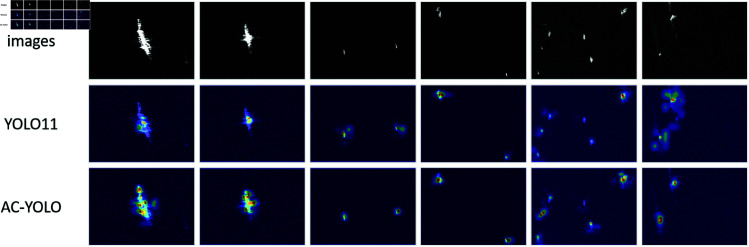
Heatmap comparison on SSDD dataset.

**Fig 11:**
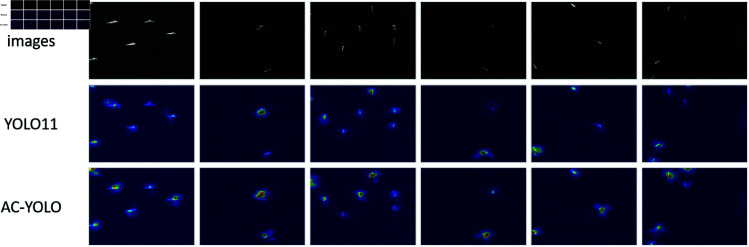
Heatmap comparison on HRSID dataset.

To intuitively demonstrate the performance improvement, we visualize the detection heatmaps of both the baseline YOLO11 model and the proposed AC-YOLO model on selected images from the SSDD and HRSID datasets in Figs 10 and 11.

As illustrated, AC-YOLO exhibits more concentrated high-activation regions (red areas) closely aligned with the true boundaries of ship targets. This indicates that the introduction of ACmix and CCFM modules significantly enhances the model’s focus on core target regions while effectively suppressing background noise. Moreover, the proposed model demonstrates superior detection accuracy and reduces false positives and missed detections compared to the baseline, underscoring its reliability.

## 5 Conclusion and future work

In this paper, we propose AC-YOLO, a lightweight SAR ship detection model tailored for complex maritime scenarios. The model effectively balances detection accuracy and computational efficiency through a series of modular innovations. To address key challenges such as small target degradation, multi-scale target coexistence, and localization errors in dense scenes, we introduce the Acmix module, which dynamically integrates self-attention and convolutional features, enabling a synergistic combination of global context modeling and local detail preservation. Additionally, CCFM module enhances multi-scale feature representation through hierarchical interactions and channel recalibration, significantly reducing model complexity while preserving detection performance. Furthermore, the MPDIoU loss function refines bounding box similarity estimation by minimizing corner-point distance, improving localization precision in densely packed scenes. Extensive experiments on publicly available datasets demonstrate that AC-YOLO outperforms state-of-the-art models in both accuracy and computational efficiency, making it a promising solution for real-time SAR ship detection, particularly in resource-constrained edge computing environments.

Despite its advantages, AC-YOLO still presents opportunities for further optimization. Future research will explore more lightweight attention mechanisms to minimize computational overhead while maintaining feature discrimination capabilities. Additionally, the current feature fusion strategy follows a fixed structure; future work could investigate adaptive fusion strategies that dynamically adjust feature extraction based on input image characteristics, enhancing adaptability to complex targets and environments. Furthermore, bounding box regression remains a critical area for improvement. Integrating gradient-aware optimization or uncertainty modeling techniques could further enhance prediction accuracy and robustness, particularly in challenging conditions such as occlusions and target overlaps.
